# Hydrogen Sulfide Removal via Sorption Process on Activated Carbon–Metal Oxide Composites Derived from Different Biomass Sources

**DOI:** 10.3390/molecules28217418

**Published:** 2023-11-03

**Authors:** Maria Baikousi, Anna Gantzoudi, Christina Gioti, Dimitrios Moschovas, Aris E. Giannakas, Apostolos Avgeropoulos, Constantinos E. Salmas, Michael A. Karakassides

**Affiliations:** 1 Department of Materials Science and Engineering, University of Ioannina, 45110 Ioannina, Greece; mbaikou@uoi.gr (M.B.); annagant99@yahoo.com (A.G.); christina.a.gioti@gmail.com (C.G.); dmoschov@uoi.gr (D.M.); aavger@uoi.gr (A.A.); 2 Department of Food Science and Technology, University of Patras, 30100 Agrinio, Greece; agiannakas@upatras.gr

**Keywords:** activated carbon, biomass, carbon composites, spent coffee, Aloe-Vera, corncob, zinc oxide, H_2_S, hydrogen sulfide, adsorption

## Abstract

Biomass exploitation is a global trend due to the circular economy and the environmentally friendly spirit. Numerous applications are now based on the use of biomass-derived products. Hydrogen sulfide (H_2_S) is a highly toxic and environmentally hazardous gas which is emitted from various processes. Thus, the efficient removal of this toxic hazardous gas following cost-effective processes is an essential requirement. In this study, we present the synthesis and characterization of biomass-derived activated carbon/zinc oxide (ZnO@AC) composites from different biomass sources as potential candidates for H_2_S sorption. The synthesis involved a facile method for activated carbon production via pyrolysis and chemical activation of biomass precursors (spent coffee, Aloe-Vera waste leaves, and corncob). Activated carbon production was followed by the incorporation of zinc oxide nanoparticles into the porous carbon matrix using a simple melt impregnation method. The synthesized ZnO@AC composites were characterized using X-ray diffraction (XRD), infrared spectroscopy (IR), thermogravimetric analysis (TGA), scanning electron microscopy (SEM), and nitrogen porosimetry. The H_2_S removal performance of the ZnO@AC composites was evaluated through sorption experiments using a handmade apparatus. Our findings demonstrate that the Aloe-Vera-, spent coffee-, and corncob-derived composites exhibit superior H_2_S sorption capacity up to 106 mg_H2S_/g_ads._, 66 mg_H2S_/g_ads._, and 47 mg_H2S_/g_ads._, respectively.

## 1. Introduction

The continuous growth of industrial processes, in combination with the increasing demand for clean energy sources, has led to a substantial rise in the emission of hazardous gases into the atmosphere. Among these harmful emissions, hydrogen sulfide (H_2_S) poses significant environmental and health risks due to its corrosive nature and toxic properties [1,2]. Anthropogenic activities such as waste landfilling, home heating, and biogas production contribute substantially to H_2_S emissions. Industrial processes, such as petroleum refining, pulp and paper manufacturing, and wastewater treatment, are some sources which release H_2_S to the atmosphere [3]. Consequently, there is a growing urgency to develop efficient and sustainable processes for H_2_S removal from industrial flue gases, biogas, and natural gas streams.

Apart from the famous but expensive and non-environmentally friendly Clauss process [4], which is still applied in oil refineries for H_2_S removal and sulfur recovery, there are several other procedures for H_2_S removal such as H_2_S absorption by amines solution [5], biological processes, oxidation, physical separation, solid-phase reactions, dry scrubbing, and chemical absorption and adsorption [3,6,7]. Each method has its own benefits and challenges as well as drawbacks, and the selection of the appropriate method depends on the specific requirements of the application, efficiency considerations, and cost-effectiveness [7].

However, among the various techniques, adsorption has emerged as the most widely applied approach for H_2_S removal due to its favorable balance between cost and effectiveness for large and small-scale applications even at low concentrations and temperatures [1,7,8]. Different adsorbent materials have been used for this purpose such as zeolites, activated carbons, and metal oxides [7]. Among these materials, activated carbons are very promising materials as effective adsorbents for H_2_S because they exhibit an interesting surface chemistry, high surface area, and tunable porosity, which enhance their sorption capacity [3,9,10]. Such materials act both as catalysts for the oxidation of H_2_S by air and as adsorbents effectively eliminating sulfur and its oxides from the fuel gas stream [10]. Moreover, biomass-derived activated carbons, which are products from carbonized biomass waste, are very attractive and environmentally friendly materials for H_2_S adsorption applications because of their natural waste origin [11,12,13,14,15,16].

In the recent years, porous carbon/metal oxide composites have emerged as promising materials for H_2_S sorption due to their unique combination of properties [17,18,19,20,21]. The incorporation of metal oxides into the carbon matrix enhances the chemical reactivity, improving the overall sorption performance. Metal oxides, such as iron oxide (Fe_2_O_3_), zinc oxide (ZnO), and manganese oxide (MnO_2_), can chemically react with H_2_S to form stable metal sulfides, increasing the overall sorption capacity and efficiency [3]. Among various metal oxides, ZnO seems to exhibit the highest equilibrium constant for sulfidation, reducing H_2_S levels to fractions of 1 ppm [22], and several studies have been reported about desulfurization using ZnO [3,23,24]. The combination of activated carbon and zinc oxide in composites could exhibit synergistic effects in H_2_S capture [25,26]. High surface area and micropore hierarchical pore structure of activated carbons, support and enhance the physical and/or chemical adsorption of H_2_S molecules. There are several publications about the proposed mechanism for H_2_S removal by adsorption in zinc oxide/alkaline activated carbon composites [27]. According to these proposed mechanisms, physisorption, chemisorption, and oxidation phenomena occur simultaneously on pores’ surfaces. In more detail, physisorption occurs according to the following reactions:

H_2_S_(gas)_ → H_2_S_(ads.)_ (1)

H_2_S_(ads.)_ → H_2_S_(ads.-liq.)_ (2)

H_2_S_(ads.-liq.)_ → HS^−^_(ads.)_ + H^+^ (3)

where H_2_S_(gas)_, H_2_S_(ads.)_, H_2_S_(ads.-liq.)_ are different phases of hydrogen sulfide.

Chemisorption occurs according to the following reactions:

H_2_S_(g)_ + KOH_(q)_-C → KHS_(q)_ + H_2_O (4)

H_2_S_(g)_ + 2KOH_(q)_-C → K_2_S_(q)_ + 2H_2_O (5)

The produced water shown in Reactions (4) and (5) covers the KOH which is impregnated in the carbon pore surface and the system KOH_(q)_-C is produced. Finally, oxidation reactions are represented with Equations (6)–(9):

HS^−^_(ads.)_ + O^*^_(ads.)_ → S_(ads.)_ + OH^−^ (6)

HS^−^_(ads.)_ + 3O^*^_(ads.)_ → SO_2(ads.)_ + OH^−^ (7)

SO_2(ads.)_ + O^*^_(ads.)_ + H_2_O_(ads.)_ → H_2_SO_4(ads.)_ (8)

H^+^ + OH^−^ → H_2_O (9)

The O^*^_(ads.)_ are adsorbed dissociative oxygen atoms and H_2_SO_4(ads.)_ is the sulfuric acid produced in reaction (8) which is responsible for the gradually decreased adsorption capacity of the activated carbon [27].

As an overall equation representative of the sorption process, the following zinc oxide exothermic chemical reaction, which leads to the chemisorption of H_2_S, is proposed:

ZnO + H_2_S ↔ ZnS + H_2_O (10)

This combined mechanism results in higher H_2_S sorption capacities and faster kinetics compared to individual components. Continued research and innovation in this field will lead to advanced technologies and will address the challenges posed by H_2_S pollution.

In this study, highly porous activated carbons derived from three different kinds of biomass waste materials (spent coffee, Aloe-Vera waste leaves, and corncob) have been investigated as effective matrices for zinc oxide incorporation and for hydrogen sulfide removal application. Inspired from Geng et al. [28], who adopted the novel melt infiltration technique to fabricate ZnO-based adsorbents, we produced novel composite ZnO@AC materials for H_2_S sorption, meeting the demands of modern times and fulfilling the criteria for simplicity, cost-effectiveness, and environmental friendliness. Overall, this study presents a promising possibility for the development of efficient H_2_S sorbents, emphasizing the significance of activated carbon/zinc oxide composites as potential candidates for mitigating H_2_S emissions in various industrial processes and environmental applications.

## 2. Results and Discussion

### 2.1. XRD Analysis of Pure AC and ZnO@AC Composites

**Figure 1:**
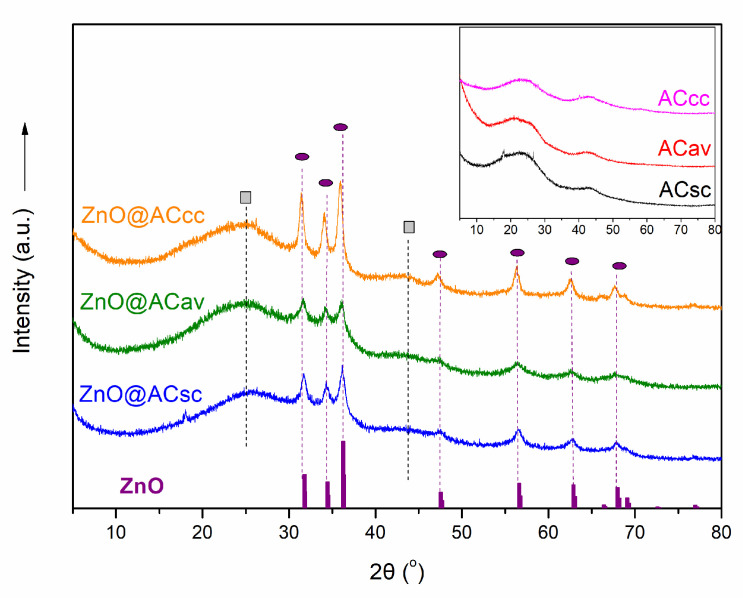
XRD patterns of ZnO@AC composites in comparison with the corresponding patterns of raw activated carbon matrices (inset figure). Grey squares correspond to activated carbon and purple circles correspond to ZnO.

An X-ray diffraction (XRD) analysis was performed on the ZnO@AC composites to investigate their structural properties and phase composition. The obtained XRD patterns are shown in Figure 1 in comparison with the patterns of the initial activated carbon matrices (Figure 1-inset). The XRD patterns of the composite materials exhibit crystalline diffraction peaks (marked with purple circles in Figure 1) due to the presence of crystalline phases of ZnO particles (JCPDS 96-230-0113) within the composite. The presence of ZnO within the activated carbon matrix is further confirmed by the overlapping of the characteristic peaks of both materials. The characteristic broad peaks at 2θ~24° and 44° (marked with grey squares in Figure 1) come from the amorphous nature of the three activated carbon matrices, as shown in Figure 1 inset. This suggests successful incorporation and dispersion of ZnO nanoparticles into the activated carbon structure. By analyzing the full width at half maximum (FWHM) of the peaks, it is possible to estimate the average crystalline size of ZnO nanoparticles within the composites using Scherrer’s equation [29,30] at 2θ~36°, which is the more intense peak for the ZnO phase. The average crystalline size for ZnO particles is calculated equal to 11, 8.3 and 13.4 nm for spent coffee, aloe leaves, and corncob ZnO@AC composites, respectively. Overall, the observed XRD patterns confirm the successful formation of ZnO@AC composite materials.

### 2.2. FTIR Spectroscopy of Pure AC and ZnO@AC Composites

**Figure 2:**
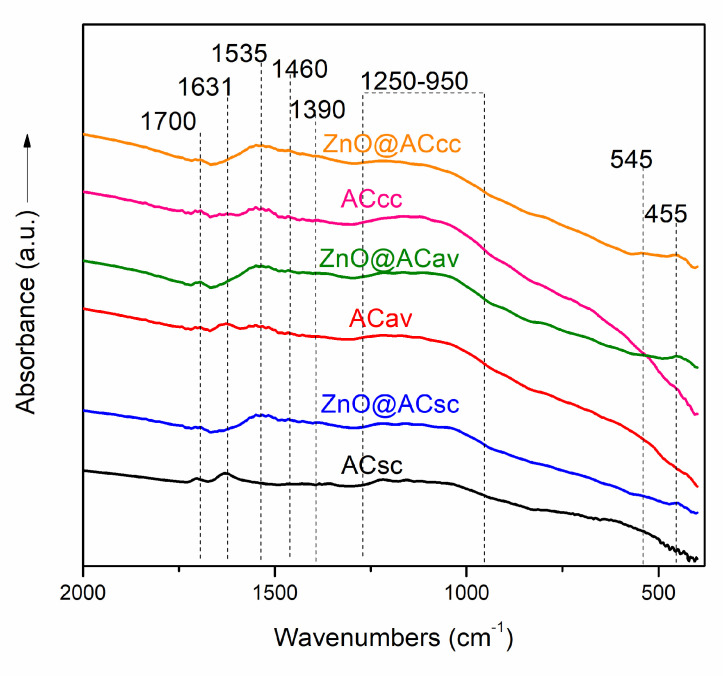
Comparison of FT-IR spectra curves of pure AC and ZnO@AC composites.

The FT-IR spectroscopy analysis was conducted to examine the chemical bonding and functional groups present in the ZnO@AC composites. The FT-IR spectra of the ZnO@AC composites (Figure 2) exhibited a combination of characteristic absorption bands from both the activated carbon and ZnO components. The bands observed in the composite spectra were compared with the individual spectra of activated carbon to identify any shifts or new band, indicating potential chemical interactions. The band observed at 1700 cm^−1^ in all spectra is attributed to the C=O stretching vibration, indicating the presence of carbonyl groups. This band may arise from the surface functional groups of activated carbon, such as carboxylic acids or ketones [31]. The absorption bands at 1460, 1535, and 1631 cm^−1^ can be attributed to the bending vibrations of CH_2_, stretching vibrations of the C=C groups in aromatic rings, and/or to the –COO^−^ groups, as well as physically adsorbed water molecules [31,32,33]. The band at 1631 cm^−1^ is shifted at 1535 cm^−1^ in the composite’s spectra, probably due to the interaction of COO^−^ groups with ZnO nanoparticles [34]. Also, in the composite’s spectra, an increase of the intensity of the weak band at 1390 cm^−1^ is observed, which could, again, be assigned to the C-OH vibration modes of activated carbon’s functional groups due to the interactions of ZnO nanoparticles with these groups [34,35]. The broad absorption band at the ~1250–950 cm^−1^ region in all spectra can be assigned to the overlapping of the C-O-O stretching, C-O stretching, and O-H bending modes of the alcoholic, phenolic, and carboxylic groups [12,36]. Furthermore, the FT-IR spectra of the composites show two weak bands at low frequency ranges, 545 and 455 cm^−1^, which can be assigned to the characteristic stretching vibration of the Zn-O bond in ZnO nanoparticles [37]. This peak indicates the successful incorporation of ZnO into the activated carbon matrix. The FT-IR analysis confirmed that the ZnO@AC composite retains the characteristic functional groups of activated carbons, indicating the coexistence of its surface chemistry with ZnO particles in the composite structure without any significant chemical degradation or transformation during the synthesis process.

### 2.3. Thermogravimetric TG% Analysis of Pure AC and ZnO@AC Composites

**Figure 3:**
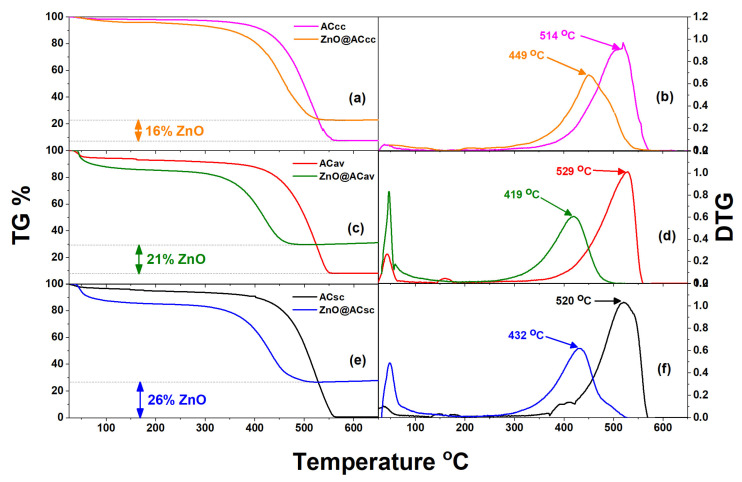
Comparison of TG% and DTG curves of pure AC and ZnO@AC composites. (**a**,**b**) Corncob biomass source, (**c**,**d**) aloe leaves biomass source, (**e**,**f**) spent coffee food waste source.

The TG% curves obtained for the ZnO@AC composites in comparison with the corresponding curves of the initial activated carbon matrices are presented in Figure 3. All TG% curves show a small initial weight loss of approximately 4–15% in the temperature range of 30 °C to 120 °C due to the removal of adsorbed moisture on the surface of the materials. A significant weight loss of approximately 55–70% is observed between 300 °C and 560 °C due to thermal degradation/combustion of activated carbon. The residual mass at this point, in the case of the ZnO@AC composite materials, is attributed to the presence of inorganic components, particularly ZnO, which has a higher thermal stability compared to activated carbon. This mass is calculated equal to 26%, 21%, and 16% for spent coffee-, aloe leaves-, and corncob-derived ZnO@AC composites, respectively. Furthermore, according to Figure 3, the ZnO@AC composites’ degradation starts earlier compared to the relevant pure AC materials. This probably happens due to the catalytic activity of the ZnO on the degradation process. Similar behavior was reported in papers for coal combustion processes with Fe_2_O_3_, CeO_2_, and CaO [38,39,40] and for enhanced photodegradation processes using ZnO nanoparticles [41,42,43].

### 2.4. Nitrogen Porosimetry for Pore Structure Analysis of Pure AC and ZnO@AC Composites

**Figure 4:**
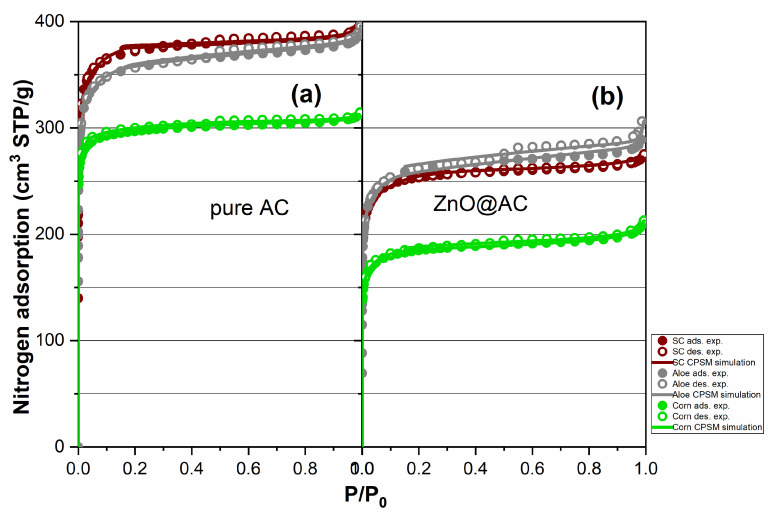
Nitrogen porosimetry experimental loops (points) of AC originating from different biomass sources i.e., spent coffee, aloe leaves, and corncob, and the relevant CPSM simulation (continuous line) (**a**) before melt impregnation with Zn salt and (**b**) after melt impregnation with Zn salt.

**Figure 5:**
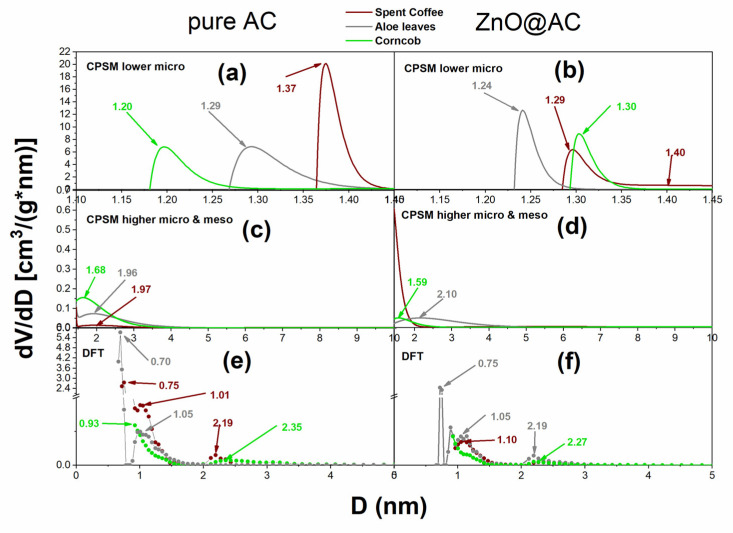
Pore volume distributions, according to CPSM and DFT models, for AC (left hand) and ZnO@AC composites (right hand), derived from different biomass sources and activated carbon composite materials. (**a**,**b**) Micro-pore region, (**c**,**d**) meso pore region, (**e**) DFT pore volume distribution for pure AC, (**f**) DFT pore volume distribution for ZnO@AC composites.

The N_2_ adsorption–desorption isotherms were measured to investigate the pore structure properties and porosity of the AC and the Zn@AC composites. The resulting hysteresis loops for all materials, as well as the simulation of the nitrogen adsorption–desorption process using the CPSM model free code downloaded from [44], are shown in Figure 4. Such results can provide us some first general results. The isotherms of all the materials are type I, showing a steep increase in adsorption at low relative pressures (P/P_0_), suggesting the existence of a mainly microporous structure [45]. There is a significant decrease in the total sorbed volume in the isotherms of Zn@AC composites in comparison with the corresponding AC matrices. However, the isotherm’s shape, which remains similar, suggests that ZnO nanoparticles did not significantly alter the textural properties of the materials and are well-dispersed within the porous framework [26]. For more detailed information, CPSM and DFT pore size distributions were obtained and presented in Figure 5. It is obvious from this figure that the two methods indicated lower micropores in the range of 1–1.5 nm. The ZnO addition seems to block slightly the very low micropores, while new microporosity is formed due to the ZnO lay down in mesopores and higher micropore structures and the relevant diameters are reduced. This could be probably an explanation for the shift of the corncob microporous distribution peak from 1.20 nm for pure AC to 1.30 nm for the ZnO@AC composite. Also, the same explanation could fit the aloe material for the shift of the microporous distribution peak from 1.29 nm for pure AC to 1.24 nm for ZnO@AC and to the spent coffee material for the shift of the microporous distribution peak from 1.37 nm for pure AC to 1.29 nm for ZnO@AC. This hypothesis is also supported by the higher micropore peak shift, which is presented in Figure 5c,d.

Numerical details for specific pore surface areas according to BET, Rouquerol, Langmuir, and the CPSM model are presented in Table 1. The negative C_BET_ values indicate a strong effect of the microporosity to the BET specific surface area calculation. This was expected because of the strong pore curvature effect in such cases, which is explained in detail in the literature [46] where calculations show that BET underestimates the specific surface close to 100%. Recently, Rouquerol et al. reported a new method for more realistic calculations of the BET surface area [47]. According to this method, higher and more realistic BET specific surface area values were estimated and are presented in the third column of Table 1, i.e., BET_Rouq._. Finally, CPSM specific surface area values which were based on CPSM pore volume distribution and are closer to the relevant values calculated using the Langmuir model seem to be more realistic also.

It is obvious from Table 1 that in all cases the underestimated specific surface areas according to the BET equation were around 1000–1100 m^2^/g when the real values seem to be around 1350–1600 m^2^/g. The ZnO addition in the AC matrix mostly affected the corncob-derived porous carbon and affected the least the aloe leave-derived porous carbon. Although in the case of pure AC the higher specific surface area belongs to the spent coffee-derived matrix, in the case of composites it belongs to aloe leaves.

The micropore fraction as well as the mean pore diameter in the low microporous region and the total pore volume of all the tested materials are presented in Table 2. The micropore fraction was estimated via two independent models, i.e., the Dubinin–Radushkevich and the CPSM model. It is obvious from such values that the majority of the pores are microporous; according to Figure 5c,d, a very small fraction is mesoporous. Furthermore, according to hysteresis loops of Figure 4, there is an extremely small fraction of macropores. The combination of these three observations could be supported by the hierarchy of the activated carbons, which is reported extensively in the literature [48,49,50]. Finally, the mean values of low micropore (i.e., from 1.0 nm to 1.5 nm) diameters is around 1.30 nm for all cases.

### 2.5. SEM-EDS Images Analysis of Pure AC and ZnO@AC Composites

**Figure 6:**
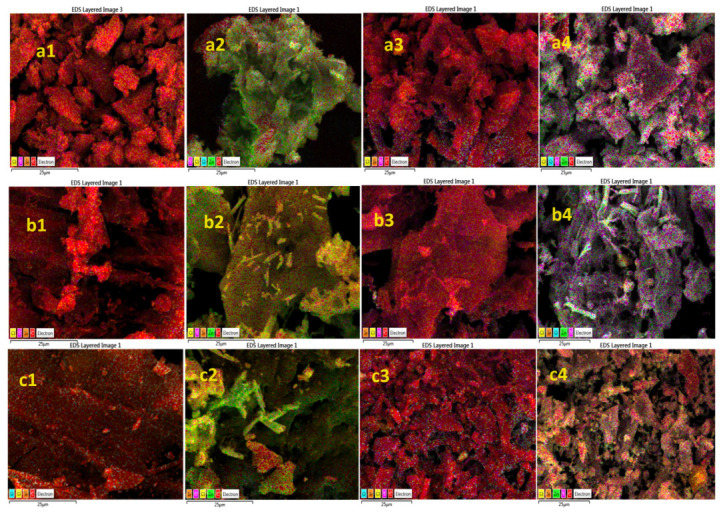
SEM-EDS mapping images for pure AC and ZnO@AC composites before and after H_2_S removal process. (**a**) Spent coffee, (**b**) aloe leaves, (**c**) corncob. (**1**) Pure AC before H_2_S removal process, (**2**) ZnO@AC composites before H_2_S removal process, (**3**) pure AC after H_2_S removal process, (**4**) ZnO@AC composites after H_2_S removal process.

Figure 6 shows SEM-EDS elemental mapping images from the AC matrices and ZnO@AC composites before and after the H_2_S sorption process, while Figure S1 displays the corresponding SEM images. It is obvious qualitatively from the change in color of Figure 6 columns 1 (pure) and 2 (composite) that the ZnO (green-yellow color) was spread homogeneously on the surface and inside the pores of the AC matrix. From the same figure, from column 4 (composite), we can observe that the sulfur (purple color) is much more abundant on the composite materials’ surface compared to the relevant, from column 3, pure AC. Thus, the composites were far more active for H_2_S removal compared to the pure AC probably because of the chemical reaction in Equation (10). Finally, from the a4 and b4 images, the spent coffee and aloe leave composites, ZnO@AC seems to be the most active materials.

**Figure 7:**
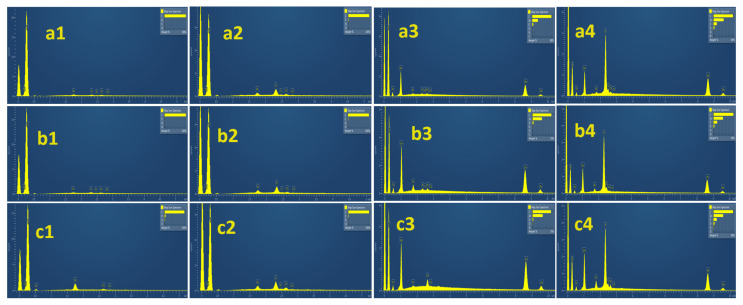
EDS quantitative analysis for pure AC and ZnO@AC composites before and after H_2_S removal process. (**a**) Spent coffee, (**b**) aloe leaves, (**c**) corncob. (**1**) Pure AC before H_2_S removal process, (**2**) pure AC after H_2_S removal process, (**3**) ZnO@AC composites before H_2_S removal process, (**4**) ZnO@AC composites after H_2_S removal process.

These results are also supported by the EDS spectrum presented quantitatively in Figure 7. It is obvious from columns 1 (pure before) and 3 (composite before) that no sulfur exists in materials before the H_2_S removal process. It is also obvious from columns 2 (pure after) and 4 (composite after) that all materials exhibit activity in the H_2_S removal process more or less. According to the inset images, pure AC materials clearly exhibit lower sulfur concentration compared to AC composites. Thus, it is confirmed that ZnO@AC composite materials are more active compared to pure AC materials.

Table 3 reports the EDS analysis values of the % wt. presence on the materials’ surface area. It is shown by the differences between fresh and used materials that sorption and probably chemical reaction processes occur in all cases. Furthermore, despite the fact that, according to Table 1, the addition of ZnO decreases the pore surface area, the H_2_S removal activity is enhanced by this addition and the aloe leave-originated composite was the most active.

### 2.6. Hydrogen Sulfide (H_2_S) Removal Experiments

Hydrogen sulfide sorption experiments were carried out using a handmade apparatus described in Section 3.5. Measurements were treated with Equations (11)–(15) and the total sorption capacity of each sample was calculated and is presented in Table 4. All AC matrices were prepared and activated via a pyrolysis process, using KOH as an activator, and under identical conditions. All ZnO@AC composites were developed via a melt-intrusion process under exactly the same conditions again. The sorption experiments were executed at ambient conditions of temperature and pressure.

Considering the column % yield, which refers to the % yield of AC using an amount of initial biomass, it is shown that corncob was the most productive source for AC. Aloe leaves were the least productive biomass probably because of the gel content which produces more gasses. Considering Table 1, the arrangement according to the specific surface area for the three sources was almost in reverse order compared to the % yield. Moreover, the H_2_S removal capacity of the pure AC originating from the corncob source is a little higher than the relevant of the spent coffee. The highest is that of pure AC produced from aloe leaves. When the porous carbon matrixes were impregnated with Zn salt and the composite ZnO@AC was formed, the composite originating from aloe leaves was again the most active material for H_2_S removal. The total capacity of 106 mg_H2S_/g_ads._ is higher than the values referring to other activated carbons and different types of ZnO composite materials (Table 5).

Also, the total capacities of the other two ZnO@AC composites, i.e., 66 mg_H2S_/g_ads._ for spent coffee and 47 mg_H2S_/g_ads._ for corncob, are remarkable. Although the specific surface was decreased because of the ZnO presence, this oxide boosted the AC activity 6.5 times in the case of spent coffee, 5.8 times in the case of aloe leaves, and 3.8 times in the case of corncob. This indicates the presence of the chemical reaction presented in the introduction section as Equation (10). It is obvious that the results concerning the H_2_S removal activity obtained from the SEM-EDS analysis agree with the results obtained from sorption experiments. Finally, even though the specific surface area and the pore structure values of the spent coffee- and the aloe leave-derived ZnO@AC composites were close, there was a gap between the respective H_2_S sorption capacities. This observation could be explained assuming a different surface chemistry effect.

## 3. Materials and Methods

### 3.1. Materials

Spent coffee (sc) was collected from the student café of the University of Ioannina and dried at 80 °C for 24 h. Aloe-Vera (av) waste leaves were supplied by the Greek Industrial Company, Hellenic Aloe, Ethnikis Antistaseos 21, Heraklion, Crete, Greece, whereas Corncob waste (cc) was supplied by Agricultural Cooperative Agrinio Union, Agrinio, Greece. Both of them were washed with tap water and dried at 80 °C for 24 h. The dried Aloe-Vera leaves and corncob were milled in order to produce fine brown powder. Potassium hydroxide (KOH, 85%), hydrochloric acid (HCl, 37%), and zinc nitrate (Zn(NO_3_)_2_·H_2_O, 98%), were purchased from Merck (Darmstadt, Germany) and used without further purification.

### 3.2. Preparation of Activated Carbons

Activated carbon (AC) was produced via the pyrolysis and chemical activation processes of spent coffee, Aloe-Vera leaves, and corncob. A certain amount of each biomass was pyrolyzed at 500 °C for 2 h under argon flow. The produced chars were ground and mixed with KOH (1/1 wt/wt) and pyrolyzed again under argon flow at 800 °C for 2 h. The pyrolysis process took place in a tubular furnace with a temperature increasing rate of 5 °C/min. The produced activated carbon was stirred for 24 h in an HCl solution (1 N), washed with deionized water to an approximately neutral pH of 7, and finally was dried at 80 °C for 24 h.

### 3.3. Preparation of ZnO@AC Composites

ZnO@AC composites were synthesized by the melt impregnation method [28] using Zn(NO_3_)_2_·H_2_O as metal precursor. An amount of 150 mg of pure AC was mixed and ground with 108 mg Zn(NO_3_)_2_·H_2_O, placed inside a 5 mL closed glass vial, and heated at 42 °C for 24 h to melt the zinc salt which was then introduced to the porous carbon. The mixture was further heated at 400 °C for 4 h under vacuum with a heating rate of 5 °C/min to produce zinc oxide molecules inside the activated carbon pores.

### 3.4. Characterization Techniques

For the chemical and structural characterization of the ZnO@AC composites, as well as of the initial activated carbon matrices, X-ray Diffraction (XRD), ThermoGravimetric analysis (TG%), Fourier Transformer–InfraRed spectroscopy (FT-IR), nitrogen (N_2_) porosimetry, Scanning Electron Microscopy (SEM), and EDX analysis were used. The XRD patterns were determined via the powder X-ray diffraction technique using a D8 Advance Bruker diffractometer equipped with a Cu Kα (40 kV, 40 mA, λ = 1.541 78 Å) radiation source and a secondary beam graphite monochromator (measuring conditions: 2 to 80° 2θ range, 0.02° steps, 2 s/step). The FT-IR spectra were collected using an FT/IR-6000 JASCO Fourier transform spectrometer in the wavenumber range of 4000–400 cm^−1^ and 4 cm^−1^ resolution. The samples were in powder form and dispersed in KBr to produce pellets. TG% analysis was performed using a Perkin Elmer Pyris Diamond TG/DTA instrument. About 5 mg of each sample was heated in air from 25 °C to 850 °C, with a temperature increasing rate of 5 °C/min. N_2_ adsorption–desorption isotherms were collected via porosimetry measurements at 77 K using a Quantachrome Autosorb iQ porosimeter. Before the analysis, the sample was outgassed at 150 °C for 20 h under a high vacuum (10^−6^ mbar). Brunauer–Emmett–Teller (S_BET_) [52], Langmuir (S_Lang._), and Corrugated Pore Structure Model (CPSM) [53,54] methods were applied to determine the specific surface area. The pore volume distribution of the material was estimated by applying the Density Functional Theory (DFT) [55] and the Corrugated Pore Structure Model (CPSM) [53,54]. The total pore volume was calculated by transforming the overall (i.e., at around P/P_0_ = 0.998) adsorbed gas nitrogen STP volume to liquid nitrogen volume. The micropore volume fraction estimations were carried out using the Dubinin–Radushkevich and the CPSM model [56,57], while the pore number (population) distribution was estimated using the CPSM model [53]. Finally, Scanning Electron Microscopy (SEM) images were obtained using a JEOM JSM 6510-LV instrument (Ltd., Tokyo, Japan) equipped with an X-Act EDS-detector from Oxford Instruments, Abingdon, Oxfordshire, UK, (an acceleration voltage of 20 kV was applied). EDX measurements were also carried out.

### 3.5. H_2_S Sorption Experimental Measurements

**Figure 8:**
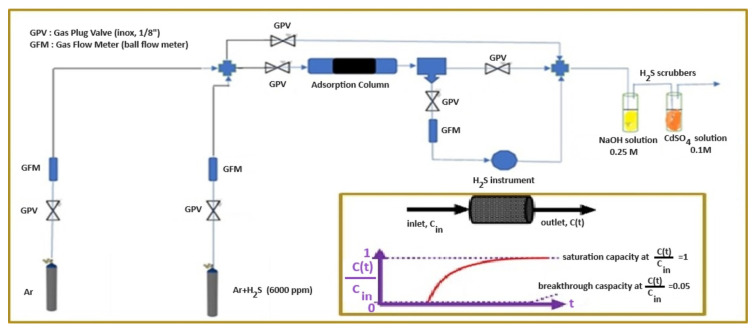
Handmade apparatus for H_2_S adsorption measurements using an artificial H_2_S + Ar gas mixture of 6000 ppm. Inset figure is a graphical representation of adsorption capacity results after mathematical treatment.

The H_2_S removal capacity of the prepared AC samples was estimated via experiments in an i.d. = 4.4 mm inox 316 adsorption column, total length of 22 mm, mounted on a handmade apparatus presented in Figure 8.

For the construction of this apparatus, we used PARKER 13HE inox 316 gas plug valves 1/8” (GPV), 172 bar, 38 °C, Parker Hannifin Sales Company, Kaarst, Germany, Pat-parker-platz 1, Kaarst, Germany, AALBORG calibrated ball gas flow meters (rotameter), Aalborg Instruments & Controls, Inc., 20 Corporate Drive, Orangeburg, NY, USA, an electrochemical instrument, CROWCON Xgard Bright, Ribble Enviro Ltd., Unit 4, Gisburn Business Park, Gisburn, Nr Clitheroe, UK for H2S sensing, and outlet concentration recording using an akYtec MSD200 4–20 mA Modbus Data Logger, GmbH, Vahrenwalder Str. 269 A, Hannover, Germany, recorder. The instrument is tuned to operate in the range of 0–6000 *v*/*v* ppm H_2_S (mL/m^3^) outlet concentration. Experiments were carried out under ambient conditions of temperature and pressure. After the adsorption column, the outlet H_2_S was firstly captured by a NaOH aqueous solution 0.25 M and secondly by a CdSO_4_ aqueous solution 0.1 M. The AC sample was placed in the adsorption column supported by glass wool in front of and behind the solid fixed bed. The length of this bed was measured before the experimental measurement started for Gas Hourly Space Velocity (GHSV) calculations. Outlet (*v*/*v*) ppm concentrations (mL/m^3^) versus time in (min) were recorded and the values were input into an excel file at the end (fully saturated AC) of each experiment. Concentration values were normalized with the maximum value, i.e., 6000 *v*/*v* ppm (mL/m^3^), and the breakthrough integral of Equation (11) and the saturation integral of Equation (12) were calculated numerically (Simpson or Trapezoid rule).

(11) $$breakthrough\;integral=I_{b}(min)=\int_{0}^{t_{b}}\left(1-\frac{C(t)}{C_{in}}\right)\times dt$$

(12) $$saturation\;integral=I_{s}\left(min\right)=\int_{0}^{t_{s}}\left(1-\frac{C(t)}{C_{in}}\right)\times dt$$

where *t_b_* (min) was the breakthrough time, i.e., time where *C*(*t*)/*C_in_* = 0.05, and *t_s_* (min) was the fully saturated time, i.e., time where *C*(*t*)/*C_in_* = 1.

The breakthrough adsorption capacity *q_b_* (mg_H2S_/g_ads._) was calculated using Equation (14), while the fully saturated adsorption capacity *q_s_* (mg_H2S_/g_ads._) was calculated using Equation (15). For such calculations, we assumed the ideality of the inlet gas and the inlet maximum concentration, i.e., 6000 *v*/*v* ppm (mL/m^3^), was converted to mg_H2S_/L_gas_, according to Equation (13).

(13) $$C_{in}\left(\frac{{mg}_{H2S}}{{ml}_{gas}}\right)=\frac{{MW}_{H2S}\left(\frac{g_{H2S}}{{mol}_{H2S}}\right)}{22400\left(\frac{{ml}_{H2S}}{{mol}_{H2S}}\right)}\times 10^{-6}\frac{m_{gas}^{3}}{{ml}_{gas}}\times C_{in}\left(\frac{{ml}_{H2S}}{m_{gas}^{3}}or\frac{v}{v}ppm\right)$$

(14) $$q_{b}\left(\frac{{mg}_{H2S}}{g_{ads.}}\right)=\frac{C_{in}\left(\frac{mgH2S}{mlgas}\right)\times \dot{V}\left(\frac{{ml}_{gas}}{min}\right)}{m_{ads.}\left(g_{ads.}\right)}\times I_{b}\left(min\right)$$

(15) $$q_{s}\left(\frac{{mg}_{H2S}}{g_{ads.}}\right)=\frac{C_{in}\left(\frac{mgH2S}{mlgas}\right)\times \dot{V}\left(\frac{ml}{min}\right)}{m_{ads.}\left(g_{ads.}\right)}\times I_{s}\left(min\right)$$

where *C_in_* is the inlet gas concentration, *MW_H2S_* is the H_2_S molecular weight, 22,400 is the molecular volume of ideal gasses, $\dot{V}$ is the gas volume flow rate, *I_b_* and *I_s_* the breakthrough and fully saturated integral respectively, *m_ads_*_._ is the adsorbent (AC) mass in the adsorption column, *q_b_* is the breakthrough adsorption capacity of the adsorbent (AC), and *q_s_* is the fully saturated adsorption capacity of the adsorbent (AC).

## 4. Conclusions

In this study, we successfully synthesized and characterized biomass-derived pure AC and ZnO@AC composites from three different biomass sources for efficient H_2_S removal. Using spent coffee, Aloe-Vera waste leaves, and corncob as biomass sources, three different activated carbon matrices were obtained by pyrolysis and chemical activation processes. Their ZnO@AC composites were also synthesized, and all the prepared materials exhibited mainly microporous structures with high specific surface area values. XRD results revealed the formation of crystalline zinc oxide particles inside the pore structure, with average crystallite sizes of 11, 8.3, and 13.4 nm for ZnO@AC derived from spent coffee, aloe leaves, and corncob, respectively. The H_2_S sorption measurements confirmed the results of the SEM-EDS analysis that the presence of ZnO significantly enhanced the material activity during this process. According to the Table 4 values, we can conclude that the biomass source for the AC production, and consequently the surface chemistry of such porous materials, plays a key role in processes such as H_2_S removal. Furthermore, as is obvious from the same table, the incorporation of ZnO with the AC matrix via a melting process significantly increases the adsorption capacity. This capacity is superior compared to other similar materials reported in Table 5.

Overall, the proposed biomass-derived ZnO@AC composites seem to be a very promising cost-effective and eco-friendly solution for H_2_S removal processes and the aloe leave-derived material, with 106 mg_H2S_/g_ads._ adsorption capacity, was the most functional for this process. Future research should focus on regeneration conditions, the effectiveness of the developed materials, and on scaling up the production of these composites and exploring their performance under real-world conditions to accelerate their practical implementation.

## Figures and Tables

**Table 1:** Specific surface areas of activated carbon matrices and ZnO@AC composite materials according to BET, Rouquerol, Langmuir, and CPSM model.

Material Code	S_g_(m^2^/g) (BET)	S_g_(m^2^/g) (BET_Rouq._)	S_g_(m^2^/g) (Lang.)	S_g_(m^2^/g) (CPSM)	Decreasing Ratio
ACsc	1195	1452	1643	1653	1.5
ZnO@ACsc	818	989	1121	1132	
ACav	1148	1498	1577	1594	1.3
ZnO@ACav	846	990	1156	1188	
ACcc	953	1210	1300	1341	1.6
ZnO@ACcc	597	726	816	832	

**Table 2:** Pore structure properties of pure AC matrices and ZnO@AC composite materials.

Material Code	Total Pore Volume (cm^3^/g)	D_mean_ (nm) CPSM Low Micro	%Microp. (CPSM)	%Microp. (Dubinin)
ACsc	0.615	1.37	91	93
ZnO@ACsc	0.422	1.29	91	93
ACav	0.613	1.29	80	90
ZnO@ACav	0.474	1.24	72	84
ACcc	0.484	1.20	82	95
ZnO@ACcc	0.329	1.30	81	87

**Table 3:** Sulfur % wt. presence on the surface of materials as it was calculated by the EDS instrument analysis.

	(SEM-EDS) S % wt.		
Material Code	Fresh	Used	Increasing Ratio
ACsc	0.32	2.39	4.84
ZnO@ACsc	0.12	10.13	
ACav	0.38	2.31	5.65
ZnO@ACav	0.23	11.13	
ACcc	0	2.46	4.05
ZnO@ACcc	0	9.97	

**Table 4:** Pure AC and ZnO@AC composites’ capacity in H_2_S removal process.

Material Code	% Yield	H_2_S Flow (mL/min)	GHSV (min^−1^)	Ads. Cap. (mg_H2S_/g_ads._)	Ads. Cap. (mmol_H2S_/g_ads._)	Times Increase
ACsc	18.9	35.7	183	10.21	0.299	6.5
ZnO@ACsc		37.4	204	66.34	1.945	
ACav	17.6	35.7	128	17.84	0.523	5.9
ZnO@ACav		35.7	136	106.03	3.109	
ACcc	23.0	35.7	286	12.42	0.364	3.8
ZnO@ACcc		35.7	217	46.90	1.375	

**Table 5:** H_2_S uptake capacities of ZnO-based adsorbents cited in the literature.

Adsorbent	Ads. Cap. (mg_H2S_/g_ads._)	Reference
AC	2.7	[26]
ZnO@AC	30.5	[26]
ZnO@N-AC	62.5	[26]
Commercial ZnO	37.7	[19]
AC	6.2	[15]
SBA-15@ZnO	18.5	[51]
SBA-15@ZnO	41.0	[28]
MCM-48@ZnO	53.2	[28]
MCM-41@ZnO	54.9	[28]

## Data Availability

The data presented in this study are available on request from the corresponding author.

## Supplementary Materials

The following supporting information can be downloaded at: https://www.mdpi.com/article/10.3390/molecules28217418/s1, Figure S1: SEM images for pure AC and ZnO@AC composites before and after H_2_S removal process.
