# Supplementary material for: Hydrogen Sulfide Removal via Sorption Process on Activated Carbon–Metal Oxide Composites Derived from Different Biomass Sources
Source: Molecules. 2023 Nov 3;28(21):7418. doi: 10.3390/molecules28217418 (PMC10650035; doi:10.3390/molecules28217418)
Supplement: Supplementary file 1 [file molecules-28-07418-s001.zip › molecules-2672444-supplementary.pdf]

Hydrogen Sulfide Removal via Sorption Process on Activated Carbon–Metal Oxide Composites Derived from Different Biomass Sources

Maria Baikousi [1], Anna Gantzoudi [1], Christina Gioti [1], Dimitrios Moschovas [1], Aris E. Giannakas [2], Apostolos Avgeropoulos [1], Constantinos E. Salmas [1,*] and Michael A. Karakassides [1,*]

Supporting Information

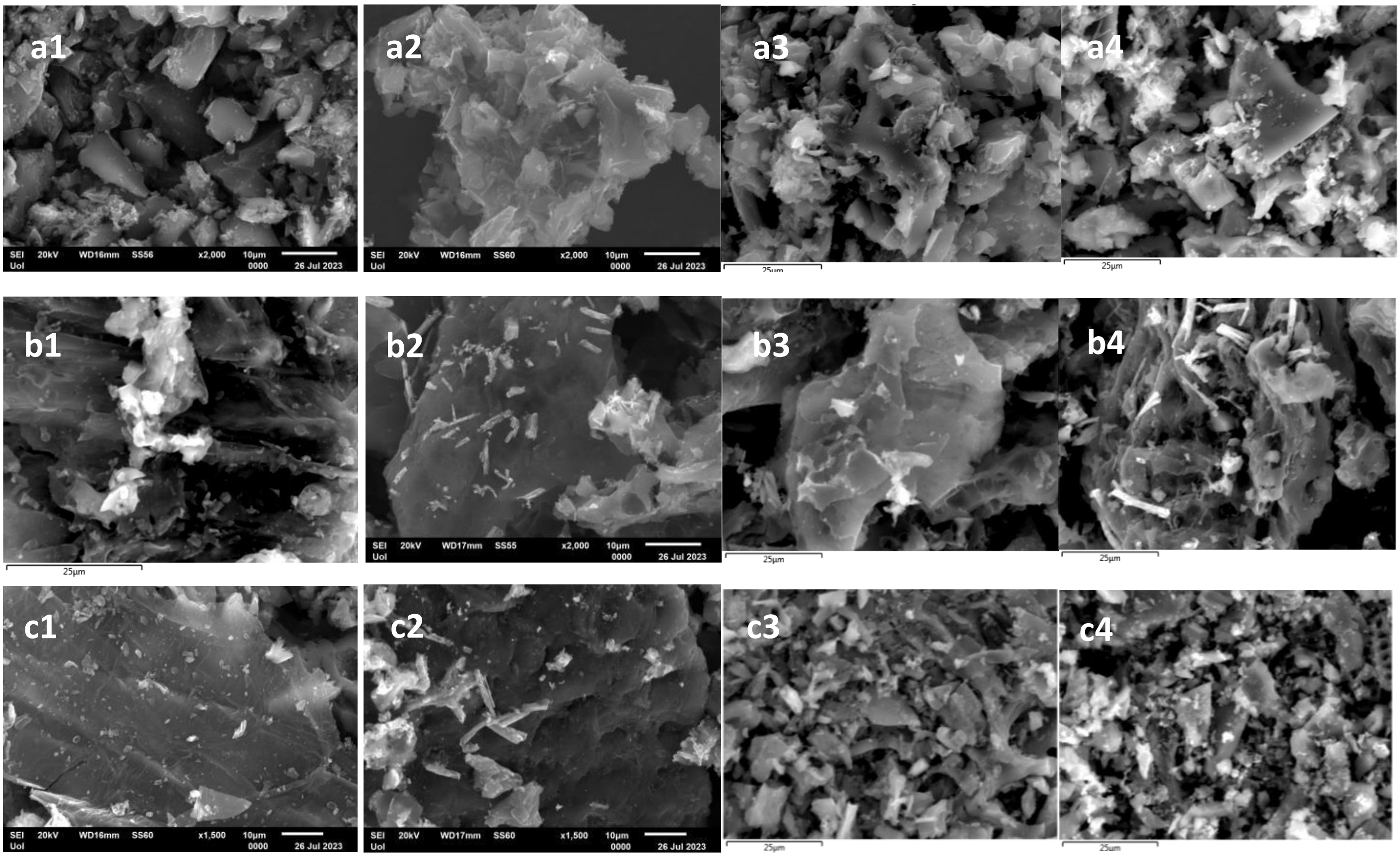

Figure S1. SEM images for pure AC and ZnO@AC composites before and after H_2S removal process. **(a)** spent coffee, **(b)** aloe leaves, **(c)** corncob. **(1)** pure AC before H_2S removal process, **(2)** ZnO@AC composites before H_2S removal process, **(3)** pure AC after H_2S removal process, **(4)** ZnO@AC composites after H_2S removal process.
